# Copy of a Letter on Typhus Fevers

**Published:** 1803-06-01

**Authors:** James Lind

**Affiliations:** Physician at Windsor, and Fellow of the Royal College of Physicians, at Edinburgh


### Copy of a Letter on Typhus Fevers, by James Lind, M.D. F.R.S. Physician at Windsor, and Fellow of the Royal College of Physicians, at Edinburgh, to Thomas Pennant, Esq. of Downing.

SIR,

THE excellent account of the diseases of the Indies, which you alluded to, is the work of the late Dr. James Lind of Haslar; but although I am not the author of that valuable book, yet I shall venture to transmit to you, a short account of what I collected on the subject *of plague in the East Indies,* during my voyages to those parts of the world.

Some physicians, with no small degree of probability, consider *jail fever* (which disease I have seen, in all its various states, in the outward bound East India fleets, during their passage from England to India) to be the same disease as the true plague that visits Turkey, Egypt, *and formerly Europe:* * This much is certain, that they agree in being communicated by contact, and being taken in by the lymphatics, occasioning buboes in the glands, into which they carry and deposit the infection. In the cases in which I have seen buboes in jail fever, they were in the axilla; whereas in Turkey they generally are in the groin,† and this I imagine, proceeds from the contagion being taken in by the lymphatics of the legs, owing to their sitting bare-legged on infected sophas. If the infection is taken by the lungs, it must be in a way that may be reckoned the same as actual contact; *viz.* by breathing the effluvia of goods *highly tainted,* ‡ or the fumes of putrid fæces from a person in the last stage of the disease; or by taking in their breath at this time; in which cases the infection is sometimes rapidly communicated to the blood,

* Sir John Pringle says, in his Annotations, Vol. viii. p. 72, which he bequeathed to the Royal College of Physicians in Edinburgh: That in August, 1770, conversing with Dr. Mackenzie, who lived eight years at Smyrna, and the last thirty at Constantinople, "I find that the common endemic pestilential fever of Constantinople, is the same as our jail or hospital fever, and they call the same distemper, when raised to a greater height, the plague; that is, when the ordinary symptoms were joined with buboes and carbuncles."

The above quotation is taken from Dr. Gardiner's Observations on the Animal Economy, p. 187.

† Cruikshank's Anatomy of the Absorbing Vessels, p. 123.

‡ Gardiner's Observations on the Animal Economy, p. 213.

blood, and the increased action of the heart and fever speedily begins.* But when it is taken by the lymphatics of the extremities, it generally requires twelve or thirteen days to reach the heart, and to begin the fever, the poison being so long retarded in passing through the lymphatics and glands of the axilla or groin.

I am confident that neither plague nor jail fever, any more than small-pox or measles, can be produced by any other means than by infection received from some person, or thing, that is tainted with their specific contagion.

In the most aggravated state that I ever saw the jail fever, in which two patients had life suspended in the first attack of the disorder, several had buboes and carbuncles,† and every symptom of true plague; yet, *by exposing those thus afflicted with it to the open air, their fevers were removed in a few days, and very few died.*

Of the many hundreds that are landed at times from the fleets from Europe, at Madrass, ill with jail fever, seldom any remains of it are to be seen, after a week or two at most, from the sick being all laid in the air, under open sheds.

It is also well known that the plague will not propagate in India, although it is said to be sometimes brought to Surat, in the cotton ships from the Red Sea, and Gulph of Persia; and although it infects a few of the people employed in unloading and repacking the bales of cotton, yet it never spreads; *owing to the inhabitants all living in a constant stream of air.*

From this cause it probably is, that the plague has never been known to rage in Hindostan; and the only fevers which induce great mortality in that part of the world, are all from marsh miasma, the *bilious putrid remitting fever.* The dysentery, undoubtedly, proceeds from the same miasma, as may hepatitis, when aided by some as yet unknown endemic contagion; the *true hepatitis* being only to be found in particular marshy situations in that country.

Among other causes which may exempt the inhabitants of Hindostan from pestilential fevers, are their daily ablutions in their sacred tanks and rivers; their anointing with oil;

---

* Gardiner's Observations on the Animal Economy, p. 241. Lind on fevers and infection, p. 33.

† The species of carbuncle here mentioned, if it may be termed a carbuncle, was hemispherical, of the size and purple-colour of an Orlean plumb, the skin very thin, and often times flaxid, and the contained liquid, a thin bloody sanious.

oil; their being altogether without cloathing, or using only those of muslin, which are washed every day. Their abstaining from animal food; their constant use of spices and fruits, and not having their floors covered with carpets, or using furs or woollen cloths, which contain contagion; a cane mat being the usual bed of an Hindoo; indeed, some few have a matt of straw, or a bit of carpet under it, and these are commonly laid in the open air.

Again, in Egypt and Turkey, where the plague is endemic, their houses are close, their chambers stifling, their floors entirely covered with carpets, and low sophas all round the rooms. Their pellices, and woollen garments, which retain and foster contagion; and the belief in predestination, which keeps them from avoiding infection, render them the promoters and victims of this deadly disorder.

While the Europeans, whose houses are commodious, well aired, and kept clean, use linen and wine in moderation, and avoid contagion as much as they possibly can, such people are but little liable to pestilential fevers. But Europe is never entirely free from pestilential contagion, it being more or less kept up in our jails, hospitals, and dirty habitations, situated in the lanes and narrow streets of cities, and crowded manufacturing towns; and from them communicated to our fleets and armies, and again by them, particularly the soldiers, disseminated over every part of the kingdom, so as to become seriously worthy of public notice.

The only possible means of diminishing this growing contagion, seems to me to be, the establishing pest-houses, or houses of recovery from pestilential fevers, in every town; and also military hospitals, for *Typhus only*, wherever there are barracks, or numbers of troops quartered, and all of them furnished with smoke and wash-houses, for the purpose of purifying the clothes of the infected, with proper hospital dresses for the patients, on the plan of those of Haslar Hospital. The impropriety of admitting typhus patients into hospitals and infirmaries, where there are convalescents from other disorders, from the *certainty* with which typhus contagion seizes them, is too well known to every medical practitioner to need being mentioned here.

